# Targeting cellular senescence prevents glucocorticoid-induced bone loss through modulation of the DPP4-GLP-1 axis

**DOI:** 10.1038/s41392-021-00528-0

**Published:** 2021-04-07

**Authors:** Tiantian Wang, Lin Yang, Zejun Liang, Lin Wang, Feijing Su, Xiangxiu Wang, Xuanhe You, Chengqi He

**Affiliations:** 1 Department of Rehabilitation Medicine, West China Hospital, Sichuan University, Chengdu, Sichuan China; 2 Key Laboratory of Rehabilitation Medicine, West China Hospital, Sichuan University, Chengdu, Sichuan China; 3 Department of MR Research Center, Nuclear Medicine, Frontiers Science Center for Disease-related Molecular Network, National Clinical Research Center for Geriatrics, West China Hospital, Sichuan University, Chengdu, Sichuan China; 4 Department of Orthopedic Surgery and Orthopedic Research Institute, West China Hospital Sichuan University, Chengdu, China

**Keywords:** Mesenchymal stem cells, Target identification, Senescence

**Dear Editor,**

Glucocorticoids (GCs) often cause detrimental side effects, including osteoporosis and osteonecrosis, with no specific drugs available to circumvent these problems.^1^ Cellular senescence plays an essential role in bone loss,^2,3^ where senescent cells release various factors, termed senescence-associated secretory phenotype (SASP), which influences neighboring cells and disrupts normal tissues.^2^ Glucocorticoids can induce senescence in bone cells in young mice;^2^ however, the effects of GCs on senescence and SASP during bone metabolism in adult mice are unclear.

Here, we firstly tested whether GC treatment could induce cellular senescence in adult mice and found that the number of lysosomal β-galactosidase (SA-βgal^+^) cells and p16INK4a^+^ cells significantly increased (Fig. 1a, c, Supplementary Fig. S1a, c) after prednisolone (Pred) treatment. However, the number of Ki67^+^ cells, osteocalcin^+^ (Ocn^+^) cells, osteoprogenitor^+^ (Osx^+^) cells, and H-type vessels, double-labeled with CD31 and endomucin (Emcn), decreased significantly after Pred injection (Fig. 1b, Supplementary Fig. S1b, d–i). Additionally, we found a noticeable decrease in bone mass in the Pred group (Supplementary Fig. S1j–o). Together, these data suggested that GCs induced cellular senescence in the metaphysis of long bones and exerted long-term detrimental effects in the bone microenvironment.

**Fig. 1:**
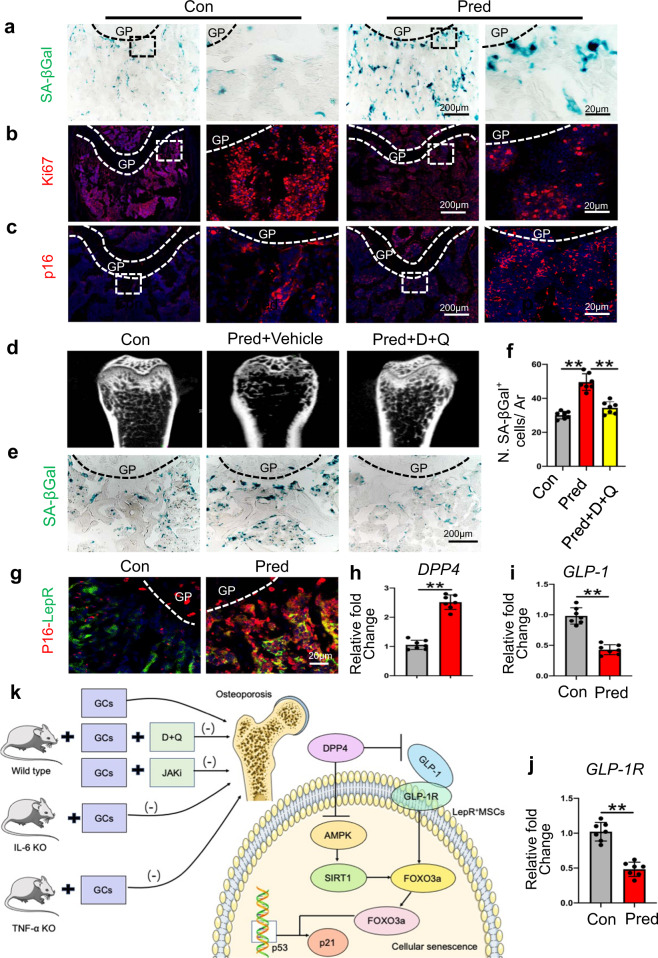
Eight-week-old C57 male mice were injected with Pred (2.5 mg/kg/d) or vehicle (**a**–**c**). Femur sections of the indicated groups were subjected to immunofluorescence staining and SA-βGal staining. Representative images of SA-βGal^+^ cells (blue) in metaphysis are shown in **a**. Femur sections of the indicated groups were subjected to immunofluorescence staining with Ki67 (red) antibodies (**b**). The sections of femurs were stained with anti-p16 antibodies (red) (**c**). Dasatinib and quercetin (D + Q) treatment (**d**–**f**). Representative micro-CT images of distal femurs (**d**, longitudinal sections). Representative SA-βGal^+^ cell staining (blue) images are shown in **e**. The number of SA-βGal^+^ cells per trabecular bone surface (N. SA-βGal^+^ cells/ Ar) is quantified in **f**. Immunofluorescence staining. Colocalization of LepR (green) with p16INK4a (red) (**g**). Analysis of DPP4 activity (**h**) and GLP-1 levels in plasma (**i**). mRNA levels of GLP-1R in LepR^+^ MSCs (**j**). GC induced LepR^+^ cell senescence through the DPP4/GLP-1 axis, modulating the AMPK-SIRT1-FOXO3a pathway. Inhibition of senescent cells or the proinflammatory secretome restores bone homeostasis in GIOP mice. GCs Glucocorticoids, TNFα KO TNFα Knockout mice, IL6 KO IL-6 Knockout mice, D + Q Dasatinib and quercetin, JAKi Ruxolitinib, GIOP glucocorticoid-induced osteoporosis (**k**). GP growth plate, Ar tissue area. DAPI was used to stain nuclei blue. *p < 0.05, **p < 0.01

Next, we determined whether the aberrant cellular senescence result in GC-induced bone loss. We treated GC-induced osteoporosis (GIOP) mice with dasatinib and quercetin (D [5 mg/kg] + Q [50 mg/kg]) once weekly to eliminate senescent cells (Supplementary Fig. S2a). As anticipated, D + Q blocked the GC-induced bone loss and reversed the increase in SA-βGal^+^ cell number in adult mice, when compared to vehicle-treated mice (Fig. 1d–f, Supplementary Fig. S2b–g). Furthermore, we found that D + Q decreased the number of p16INK4a^+^ cells and rescued Ki67^+^ cells (Supplementary Fig. S2h, i, m, n), H-type vessels (Supplementary Fig. S2j, o), and osteoblasts (Supplementary Fig. S2k, l, p, q). These findings suggested that clearance of senescent cells could reverse bone loss induced by GCs.

To examine whether clearing SASP in GC-treated mice influenced bone remodeling, we treated GIOP mice with the anti-inflammatory drug ruxolitinib (JAKi). Moreover, there was a decrease in senescent cells but an increase in Ocn^+^ cells and H-type vessels (Supplementary Fig. S3a–f) after D + Q treatment. JAKi-treated mice also preserved the trabecular-bone microarchitecture within the femur (Supplementary Fig. S3g–m). A dramatic decrease in Ki67^+^ cells, along with an increase of p16INK4a^+^ cells, after Pred treatment in the Con group was not seen in the TNF-α knockout (TNF-α KO) or IL-6 knockout (IL-6 KO) mice (Supplementary Fig. S4a, b, e, f). Furthermore, there were more Osx^+^ cells and H-type vessels (Supplementary Fig. S4c, d, g, h) in these gene-knockout mice when compared to those in GIOP mice. Consequently, an improvement in bone phenotype in the TNF-α KO and IL-6 KO mice treated with GCs was observed when compared to that in the GIOP mice (Supplementary Fig. S4i–m). These findings suggest that inhibition of pro-inflammatory cytokines suppresses cellular senescence and attenuates bone loss caused by GCs. Notably, GCs are known to inhibit proinflammatory cytokine production, and thererfore, there may exist a dose response for GCs, which may be contributing to the paradoxical effects seen on SASP in bone.

The leptin receptor (LepR) is a marker that highly enriches bone mesenchymal stem/stromal cells (MSCs) during bone remodeling in adults.^4^ We found an increase in co-staining of p16INK4a-LepR, with almost 73% of p16INK4a^+^ cells exhibiting an increased DNA damage burden (Fig. 1g, Supplementary Fig. S5a–c). We also found that mRNA levels of the senescent and SASP markers were markedly increased after GC treatment (Supplementary Fig. S5d–j). Together, these results suggest that GC treatment leads to cellular senescence of LepR^+^ cells in SASP.

Dipeptidyl peptidase-4 (DPP4) plays an important role in a variety of diseases, such as musculoskeletal disorders. We found that the activity of DPP4 and its protein expression levels increased after GC treatment (Fig. 1h, Supplementary Fig. S6a). Moreover, GCs upregulated the protein expression of p53, whereas an inhibitor of DPP4, saxagliptin, or a targetted SiRNA (Supplementary Fig. S6b, c) significantly inhibited the effects of GC treatment. These data indicate that upregulation of DPP4 signaling in LepR^+^ cells may contribute to GC-induced cell senescence. DPP4 can degrade GLP-1, which can attenuate endothelial senescence,^5^ and we found a reduction in GLP-1 levels in plasma, as well as a decrease in GLP-1 receptor (GLP-1R) levels in LepR^+^ cells after GC treatment (Fig. 1i, j). Western blot results showed that GC treatment upregulated p53 expression, whereas exendin 4, an agonist of the GLP-1R, blocked the effects of DPP4 treatment (Supplementary Fig. S6d). Moreover, exendin 9-39, an inhibitor of the GLP-1R, abolished the protective effect of saxagliptin on GC-induced cellular senescence (Supplementary Fig. S6e). These data suggest that the senescent effect of GCs is DPP4/GLP-1 dependent. Next, we co-treated animals with GC and sitagliptin (an inhibitor of DPP4) and as can be seen in Supplementary Fig. S6f-i, sitagliptin was able to suppress the number of SA-βGal^+^ cells, but increase the number of Ki67^+^ cells. Furthermore, GCs caused a low-bone-mass phenotype (Supplementary Fig. S7a-f) and this effect could be reversed by sitagliptin. In addition, sitagliptin also blocked the suppressive effects of Pred on osteogenesis and angiogenesis (Supplementary Fig. S7g–l). QT-PCR analysis showed that while Pred decreased GLP-1R expression and enhanced p16INK4a and p21 levels, co-treatment with sitagliptin significantly attenuated these changes (Supplementary Fig. S6j–m). Generally, therefore, systemic administration of sitagliptin protected against the negative effects of GC treatment.

Here we attempted to identify the molecular mechanisms involved in GC-induced senescence by looking at the role of DPP4 inhibition on SIRT1 expression and its upstream regulator, AMP-activated protein kinase-α (AMPKα). As observed using in vitro cell culture, SIRT1 levels, AMPKα expression, and the phosphorylation status of AMPKα were all reduced in response to Pred treatment in senescent LepR^+^ cells. However, saxagliptin, or inhibition with a SiRNA, significantly reversed these changes (Supplementary Fig. S8a, b). Therefore, SIRT1, AMPKα, and the phosphorylation level of AMPKα could be restorded by DPP4 inhibition after Pred treatment. The expression of p53 was increased in the cells treated with Ex527, the SIRT1 inhibitor, relative to the cells transfected with SiDPP4 or treated with the DPP4 inhibitor (Supplementary Fig. S8c, d). In contrast, SIRT1 inhibition did not induce any effect on AMPKα expression or its phosphorylation level (Supplementary Fig. S8e). We noted that Forkhead box O3 (FOXO3a) was decreased by GCs, but reversed by DPP4 inhibition and SIRT1 activation (Supplementary Fig. S8f, g), suggesting a complex relationship between p53 and FOXO3a. Knockdown of FOXO3a induced changes to the subcellular localization of p53, leading to its decreased nuclear exclusion in LepR^+^ cells (Supplementary Fig. S9a). Cytosolic p53 levels reduced by FOXO3a SiRNA, therefore, might contribute to the decrease in the total protein level of p53 (Supplementary Fig. S9b). Additionally, we found that silencing FOXO3 reduced the level of phosphorylation seen at Serine-15, which is related to DNA damage (Supplementary Fig. S9b). Collectively, our results suggested that GC-induced LepR^+^ cell senescence is working through the AMPKα/SIRT1/FOXO3a pathway.

In our current study, we found that administration of GCs induced senescence of LepR^+^ cells and that inhibition of senescent cells or the proinflammatory secretome restores bone homeostasis in GIOP mice. Moreover, we found that the DPP4 /GLP-1 axis and AMPK/SIRT1/FOXO3a pathway may be involved in GC-induced cellular senescence, thereby offering insights into the development of novel potential therapies for GIOP (Fig. 1k).

## Supplementary information

SUPPLEMENTAL MATERIAL

## Data Availability

Additional data collected during this study are available from the corresponding author upon reasonable request.
