# Supplementary material for: Targeting cellular senescence prevents glucocorticoid-induced bone loss through modulation of the DPP4-GLP-1 axis
Source: Signal Transduct Target Ther. 2021 Apr 7;6:143. doi: 10.1038/s41392-021-00528-0 (PMC8024331; doi:10.1038/s41392-021-00528-0)
Supplement: Supplementary file 1 — SUPPLEMENTAL MATERIAL [file 41392_2021_528_MOESM1_ESM.docx]

**Supplementary Materials for**

Targeting cellular senescence prevents glucocorticoid-induced bone loss

through modulation of the DPP4-GLP-1 axis

Tiantian Wang^1^, Lin Yang^1^, Zejun Liang ^1^, Lin Wang^1^, Feijing Su^2^, Xiangxiu Wang^1^, Xuanhe You^3^, Chengqi He^1^*,

Correspondence to: hxkfhcq2015@126.com

**This PDF file includes:**

Materials and Methods

Figures. S1 to S10

**Materials and Methods**

**Animals and drug treatment**

Eight-week-old C57BL/6 (WT), TNFα-/- (heterozygotes), and IL-6 -/- (heterozygotes) male mice were assigned to six groups of ten animals each, including: control mice (saline injection) from each genotype; Pred (prednisolone injected) mice from each genotype. All animals were housed in an animal facility with the consent of the Institutional Animal Care and Use Committee at the West China Hospital, Sichuan University, and maintained in a temperature controlled facility with an exact 12 h light/dark cycle and given a normal chow diet and water ad libitum. One group from each genotype was injected with 2.5 mg/kg/d Pred to guarantee an effect from GCs up to 4 weeks, thereby inducing osteoporosis; the others were injected with normal saline as the controls. All animals were sacrificed for blood and bone analyses. After stripping the skin, the femurs were fixed in 4% paraformaldehyde for micro-CT and histological staining. PCR analyses was appiled to determin genotypes, using oligonucleotide primer sequences directed toward the mutated TNFα and IL-6 genes according to the Jackson Laboratory.

Dasatinib and quercetin (D+Q) treatment: Eight-week-old C57BL/6 male mice were randomly assigned to three groups: co-treatment of GC, once weekly by oral gavage with D+Q for 1 month; or co-treatment with GC and vehicle for 1 month; or the control group. D (dasatinib, Selleck Chemicals, 5 mg/kg) and Q (quercetin, Selleck Chemicals, 50 mg/kg) were delivered by oral gavage.

The JAK1/2 inhibitor: Ruxolitinib (JAKi) (INCB018424, Selleck Chemicals, Houston, TX, USA) was used. The mice were randomly assigned to three groups: control group; co-treatment with GC and JAKi; or co-treatment with GC and vehicle for 1 month. The mice were fed JAKi (0.5 g mixed with chow) each day for 1 month including a dose of 120 mg/kg ruxolitinib (drug/body weight).

The DPP4 inhibitor (DPP4i), sitagliptin (MK-0431, Selleck Chemicals, Houston, TX, USA) at a concentration of 4 g/kg mixed in chow. Eight-week old mice were randomly assigned to a contol (Con) group, co-treatment with GC and sitagliptin for 1 month group, or co-treatment with GC and vehicle for 1 month group.

**Micro-computed tomography (micro-CT)**

Micro-CT analysis applied (Viva CT80, Scanco Medical, Switzerland) with the following parameters: 70 kV, 114 mA, and 700 ms integration time. Trabecular measurement scanning continuously acquired 100 micro-CT images, with a layer spacing of 10 μm. Sanco software version 5.0 was used to construct complete femoral metaphyseal three-dimensional (3D) structures. Analysis parameters included: bone volume/tissue volume (BV/TV, %), trabecular thickness (Tb.Th, mm), separation (Tb.Sp, mm), number (Tb.N, mm^-1^) and cortical bone thickness (Ct. Th, mm).

**Immunohistochemistry and immunofluorescence staining of bone tissue sections**

After sacrifice, adherent muscle was removed from right femurs, which were fixed in the 4% paraformaldehyde solution overnight and then decalcified in 20% EDTA buffer for 21 days. After decalcification, immunofluorescence staining was performed by immersing the bones in 20% sucrose and 2% polyvinylpyrrolidone solution for 24 hours. Finally, the tissues were embedded in OCT, and SA-βgal was detected as a marker to identify senescent cells, per to manufacturer’s instructions (Cell Biolabs, CBA-230). Sections were incubated with primary antibodies to Ki67 (Abcam, ab16667), osterix (Abcam, ab22552), osteocalcin (Takara, M188), Emcn (Santa Cruz Biotechnology, sc-65495), CD31 (R&D Systems, FAB3628G), p16INK4a (Santa Cruz Biotechnology, sc-1661), DPP4 (Abcam, ab28340), Gamma-H2AX (Abcam ab26350,) and LepR (R&D Systems, BAF497) overnight at 4 °C. All secondary antibodies were purchased from (Thermo Fisher Scientific). DAPI stains nuclei blue. Images were taken using a Zeiss LSM880 and Nikon (N-STORM & A1 Ti2) confocal microscope.

**Cell sorting and flow cytometry analysis of LepR^+^ cells**

After sacrifice, collected bone marrow cells from femurs and tibias. ACK lysis buffer (Gibco, A1049201) was used to remove red blood cells. Then incubated with primary antibody on ice in the dark for 30 min. The primary antibodies included: LepR (R&D Systems, BAF497, 1:100) and PerCP cy5.5-conjugated anti-mouse CD45 (Becton Dickinson). Incubated with secondary antibody for 30 mins, washed with PBS solution three times, and then transferred to flow tubes. Cells sorting was then performed on a 6-laser BD FACS system, based on a CD45 negative and LepR positive sorting strategy (Becton Dickinson).

**Quantitative real-time PCR**

Total RNA was extracted from both the sorted cells and bone tissue for qRT-PCR analysis using Trizol reagent, according to the manufacturer’s instructions (Invitrogen, CA, USA) Absorbence readings at A260/A280 were then used to evaluate RNA quality. PrimeScript RT reagent kit (Takara Bio, Japan) was used to prepare cDNA. Real-time quantitative PCR was performed with a SYBR Premix Ex Taq II kit (Takara Bio, Japan) in an iCycler (Bio-Rad) real-time PCR machine, according to the manufacturer's instructions. The relative mRNA expression levels were calculated using the 2^-△△CT^ method, with GAPDH for normalization.

**Western Blot Analysis**

Cold PBS was used to wash cells which would then be lysed in RIPA lysis buffer. The solution was then centrifuged and total protein could be extracted from the supernatant. 10% SDS-PAGE were used to separate proteins (20 μg per sample) which would then be blotted onto polyvinylidene fluoride membranes. Fat-free milk (5%) would be used to block the membranes for 1 hour in room temperature, followed by incubation using primary antibodies at 4°C. The next day, the membranes were washed in TBST solution, and then incubated with secondary antibodies for 1h at room temperature. Finally, enhanced chemiluminescence detection reagents were used to visualize the protein bands of interest. The primary antibodies used were against: DPP4 (Abcam, ab28340), AMPKα (Cell Signaling Technology, 2532), p-AMPKα (Cell Signaling Technology, 2535), SIRT1 (Abcam, ab110304), FOXO3a (Cell Signaling Technology, 2497) and GAPDH (Abcam, ab8245).

**Statistical Analysis**

All experiments were performed at least three times. All results are expressed as mean ± s.e.m. Statistical analysis was performed with one-way ANOVA for multiple comparisons and two-tailed Student's t-tests for comparisons between 2 groups. p< 0.05, p< 0.01 were deemed statistically significant.

**
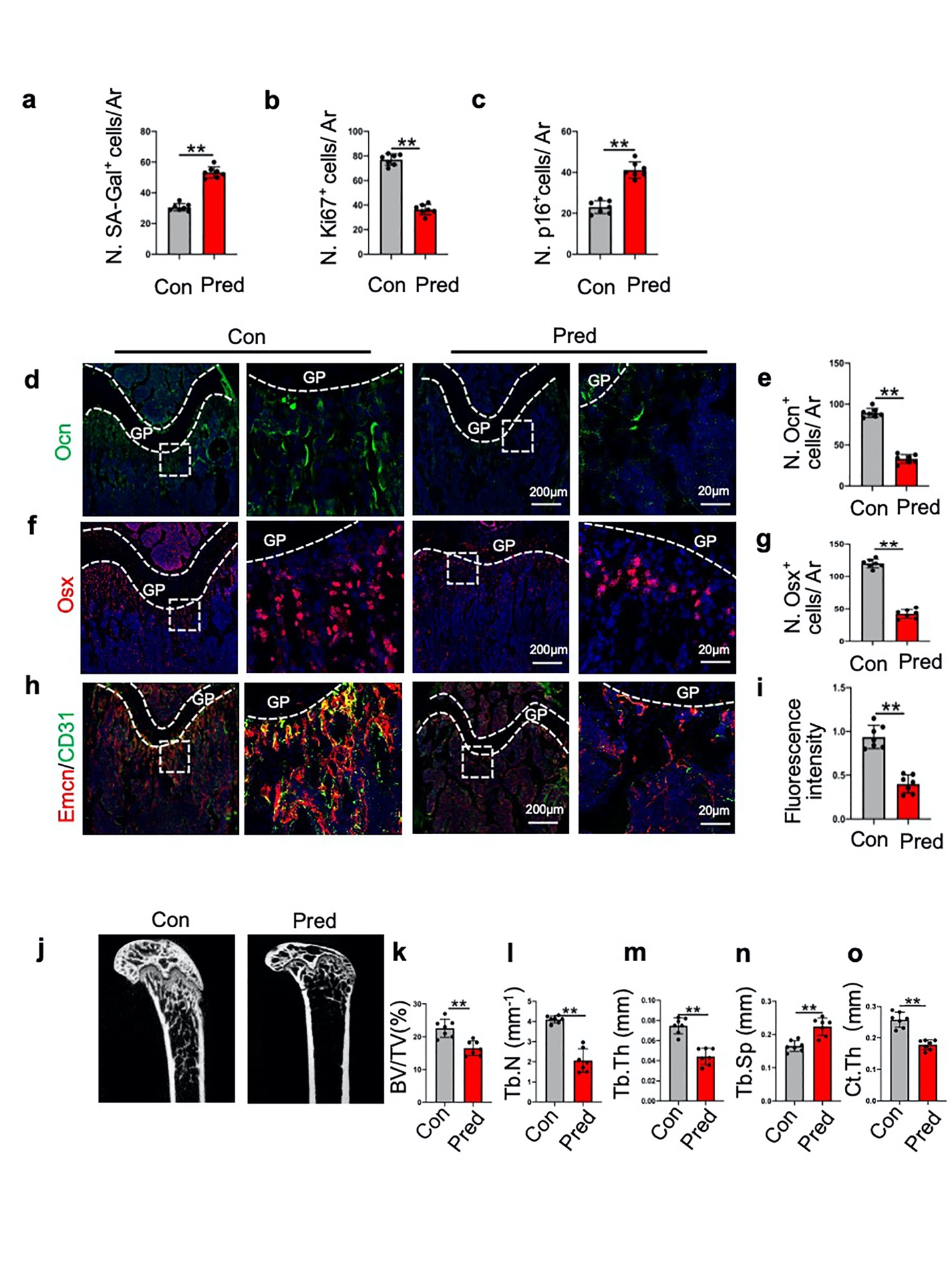
**

**Supplementary Fig. S1 Association of accumulated senescent cells in GIOP**

Eight-week-old C57 male mice were injected daily with Pred or vehicle intraperitoneally. Femur sections of the indicated groups were subjected to immunofluorescence staining and SA-βGal staining. Number of SA-βGal^+^ cells per trabecular bone surface (N. SA-βGal^+^ cells/ Ar) (a). Number of Ki67^+^ cells per trabecular bone surface (N. Ki67^+^ cells/ Ar) (b). Quantification of p16INK4a^+^ cells in femoral primary spongiosa per mm^2^ tissue area (N. p16INK4a^+^cells/ Ar) (c). Femur sections of the indicated groups were subjected to immunofluorescence staining (d, f, h). Representative confocal images are shown in (d). Green: osteocalcin (Ocn^+^) cells; blue: nuclear staining using DAPI. Number of Ocn^+^ cells per trabecular bone surface (N. Ocn^+^ cells/ Ar) (e). Representative confocal images are shown in (f). Red: osterix (Osx^+^) cells; Blue: nuclear staining by DAPI. Quantified numbers of Osx^+^ cells per trabecular bone surface (N. Osx^+^ cells/ Ar) (g). Representative images of H-type vessels (characterized by high costaining for CD31 [green] and endomucin [Emcn] [red]) are shown in (h). Quantitfication of relative fluorescence intensities in femoral metaphysis (i). Representative micro-CT images of distal femurs (j). Quantitative analyses of trabecular bone volume fraction (BV/TV) (k), trabecular number (Tb.N) (l), trabecular thickness (Tb.Th) (m), trabecular separation (Tb.Sp) (n) and cortical thickness (Ct.Th) (o). Seven mice per group were used and data given as mean ± s.e.m. GP, growth plate; Ar, tissue area. DAPI was used to stain nuclei blue. *p< 0.05, **p< 0.01; (Student’s t-test).

**
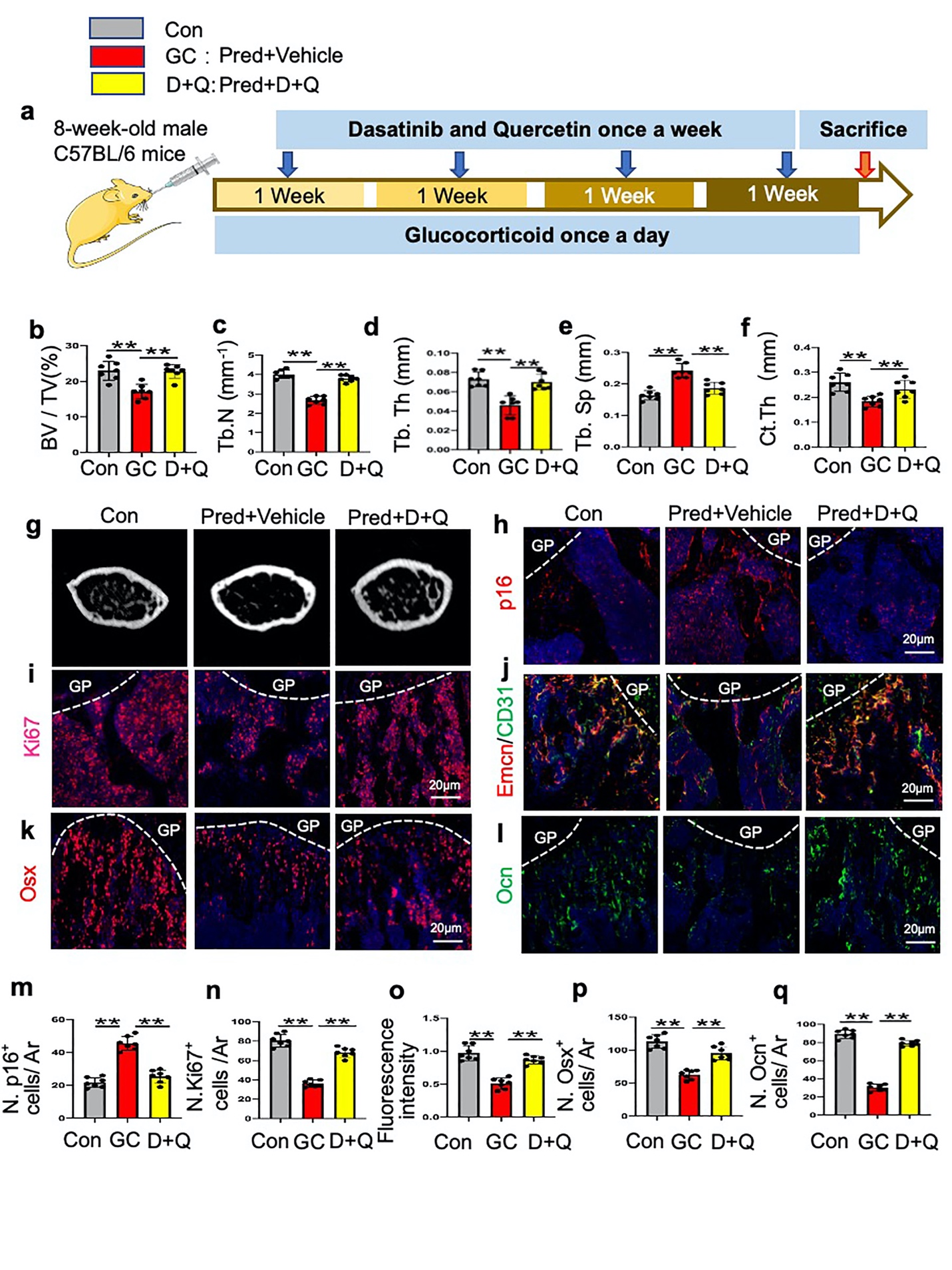
**

**Supplementary Fig. S2 Clearance of senescent cells by dasatinib and quercetin preserves bone loss in GIOP**

Dasatinib and quercetin (D+Q) treatment (a-q). Schematic diagram illustrating the experimental procedure (a). Quantitative analyses of trabecular bone volume fraction (BV/TV) (b), trabecular number (Tb.N) (c), trabecular thickness (Tb.Th) (d), trabecular separation (Tb.Sp) (e) and cortical thickness (Ct. Th) (f). Representative micro-CT images of distal femurs (g,cross sections). Immunofluorescence staining (h-l). Immunostaining of p16INK4a (red) (h), Ki67 (red) (i), as well as CD31 (green)-endomucin (Emcn) (red) (j). Number of p16INK4a^+^cells (N. p16INK4a ^+^ cells/ Ar) and Ki67^+^cells (N. Ki67^+^ cells/ Ar) in primary spongiosa per mm^2^ tissue area are shown in (m) and (n), respectively. Quantification of relative fluorescence intensities in primary spongiosa (o). Representative images of osterix^+^(Osx^+^) cells (red) and osteoclain^+^ (Ocn^+^) cells (green) in longitudinal femur sections are shown in (k) and (l), respectively. Number of Osx^+^ cells (N. Osx^+^ cells/ Ar) and Ocn^+^cells (N. Ocn^+^ cells/ Ar) per trabecular bone surface are shown in (p) and (q), respectively. GP, growth plate; Ar, tissue area. Nuclei were stained by DAPI. Seven mice per group were used and data are shown as mean ± s.e.m. *p< 0.05, **p< 0.01; (One-way ANOVA).

**
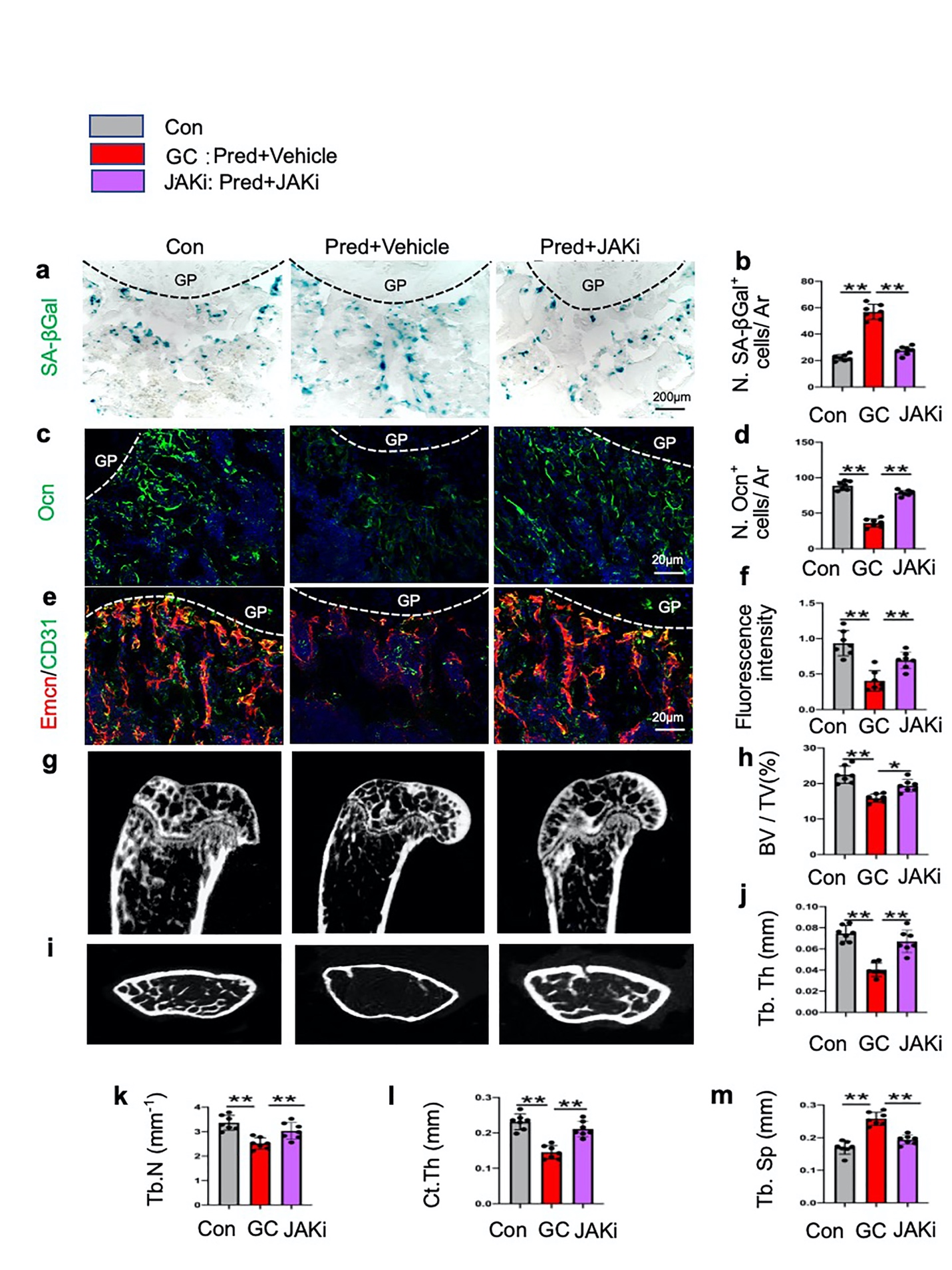
**

**Supplementary Fig. S3 Suppressing SASP by treatment with the JAK1/2 inhibitor ruxolitinib prevents GC-induced bone loss**

Ruxolitinib (JAKi inhibitor) treatment (a-m). Representative SA-βGal staining (blue) images are shown in (a). Quantification of SA-βGal^+^ cells per trabecular bone surface (N. SA-βGal^+^ cells/ Ar) (b). Representative images of immunofluorescence staining of osteocalcin (Ocn) (green) (c). Numbers of Ocn^+^ cells per trabecular bone surface (N. Ocn^+^ cells/ Ar) are shown in (d). Representative images of CD31 (green) and endomucin (Emcn) (red) are shown in (e). Quantification of relative fluorescence intensities in femoral metaphysis (f). Representative micro-CT images of distal femurs in male mice are shown in (g, longitudinal sections) and (i, cross sections). Quantitative analyses of bone volume/tissue volume (BV/TV) (h), trabecular number (Tb.N) (k), trabecular thickness (Tb.Th) (j), cortical thickness (Ct. Th) (l) and trabecular separation (Tb.Sp) (m). GP, growth plate; Ar, tissue area. DAPI was used to stain nuclei blue. Seven mice per group were used and data shown as mean ± s.e.m. *p< 0.05, **p< 0.01; (One-way ANOVA).

**
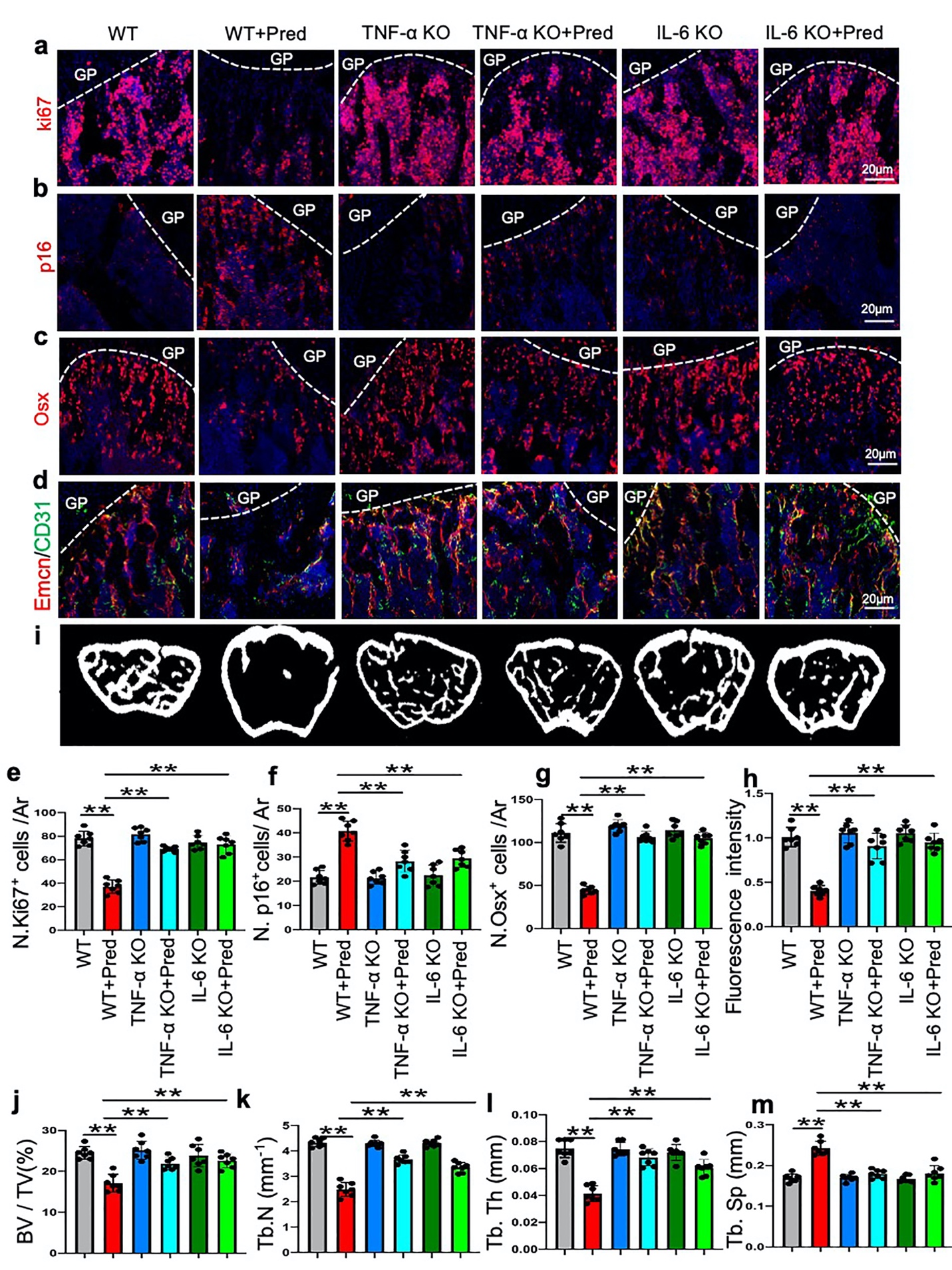
**

**Supplementary Fig. S4 Deletion of TNFα and IL6 antagonized the effects of GCs on bone**

Eight-week-old TNF-α knockout mice (TNF-α KO) and IL-6 knockout mice (IL-6 KO) were injected with Pred or vehicle. Representative confocal images are shown in (a and b). Red: Ki67^+^ or p16^+^cells; Blue: nuclear staining by DAPI. Number of Ki67^+^ (N. Ki67^+^ cells/ Ar) and p16INK4a ^+^ cells (N. p16INK4a ^+^ cells/ Ar) per trabecular bone surface are shown in (e) and (f), respectively. Representative images of osterix^+^ (Osx^+^) cells (red) in longitudinal femur sections (c). Number of Osx^+^ cells in primary spongiosa per mm^2^ tissue area (N. Osx ^+^ cells/ Ar) (g). Representative images of H type vessels (characterized by high costaining for CD31 [green] and endomucin [Emcn] [red]) (d). Quantification of relative fluorescence intensities in femoral metaphysis (h). Representative micro-CT images of distal femurs are shown in (i). Quantitative analyses of trabecular bone volume/tissue volume (BV/TV) (j), trabecular number (Tb. N) (k), trabecular thickness (Tb. Th) (l), trabecular separation (Tb. Sp) (m). GP, growth plate; Ar, tissue area. DAPI was used to stain nuclei blue. Seven mice per group were used and data shown as mean ± s.e.m. *p< 0.05, **p< 0.01; (One-way ANOVA).

**
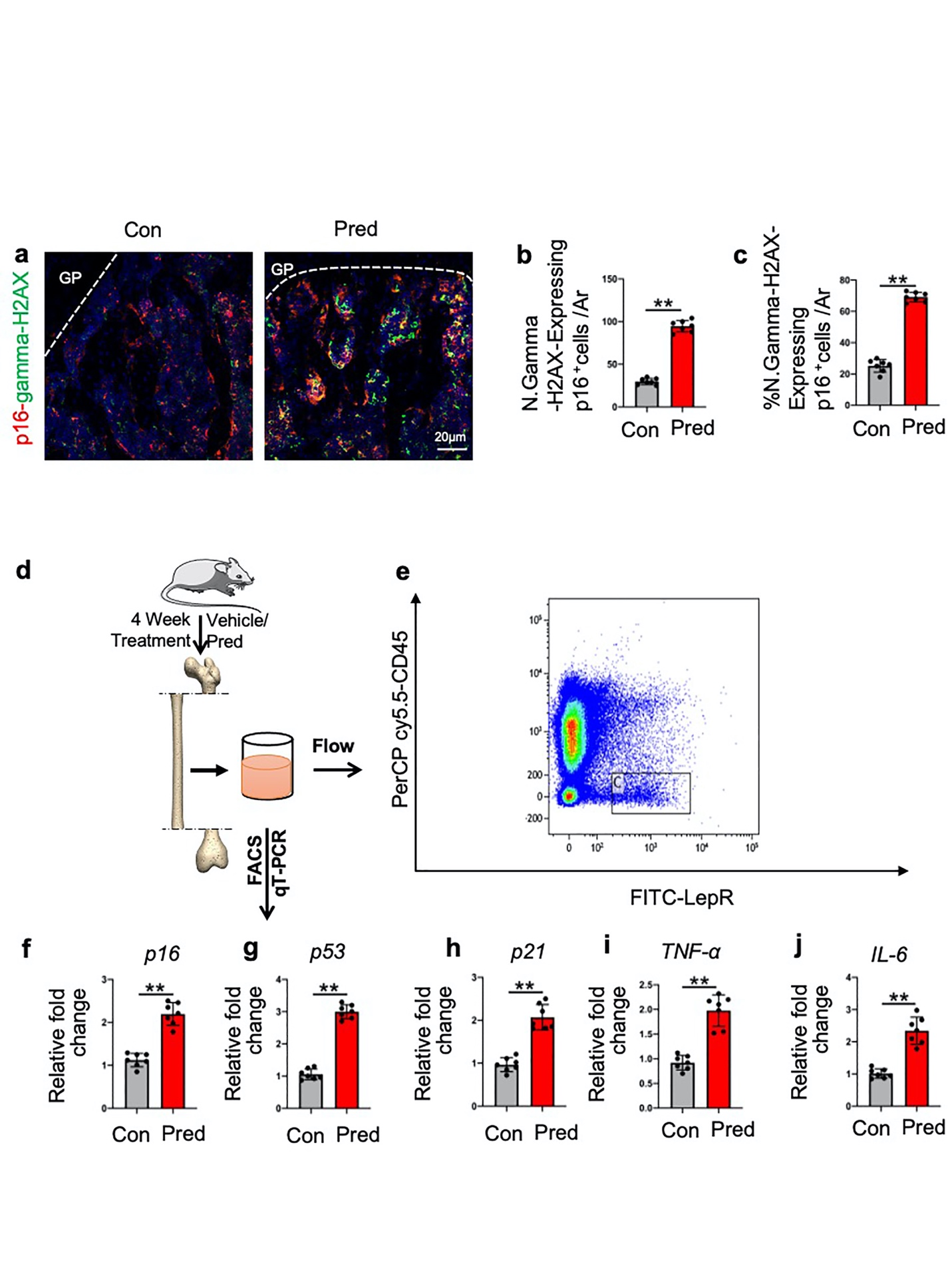
**

**Supplementary Fig. S5 GCs induce senescence of LepR^+^ cells and develop SASP**

Eight-week-old WT mice were injected with Pred at 2.5 mg/kg/day or vehicle. Colocalization of Gamma-H2AX (green) with p16INK4a (red) in femoral metaphysis (a). Nuclei were stained by DAPI. Quantification of the number (N. Gamma-H2AX-Expressing p16^+^ cells/ Ar) and percentage (% N. Gamma-H2AX-Expressing p16INK4a^+^ cells/ Ar) of gamma-H2AX^+^-p16INK4a^+^ cells in femoral metaphysis sections per mm^2^ tissue area are shown in (b) and (c), respectively. Schematic diagram illustrating the experimental procedure (d). Isolated cells were flushed from femurs after cutting the end of the bones, and the isolated cells were subjected to flow cytometry analysis. Representative flow cytometry plots showing targeted cells sorted from bone marrow cells based on a CD45 negative and LepR positive sorting strategy (e). The cell suspensions were prepared as described in (d) and were subjected to FACS sorting followed by qRT-PCR analysis. Quantitative real-time analysis of the mRNA expression of p16INK4a (f), p53 (g), p21 (h), TNF-α (i), and IL-6 (j). Seven mice per group were used and data shown as mean ± s.e.m. GP, growth plate; Ar, tissue area. *p< 0.05, **p< 0.01; (Student’s *t*-test).

**
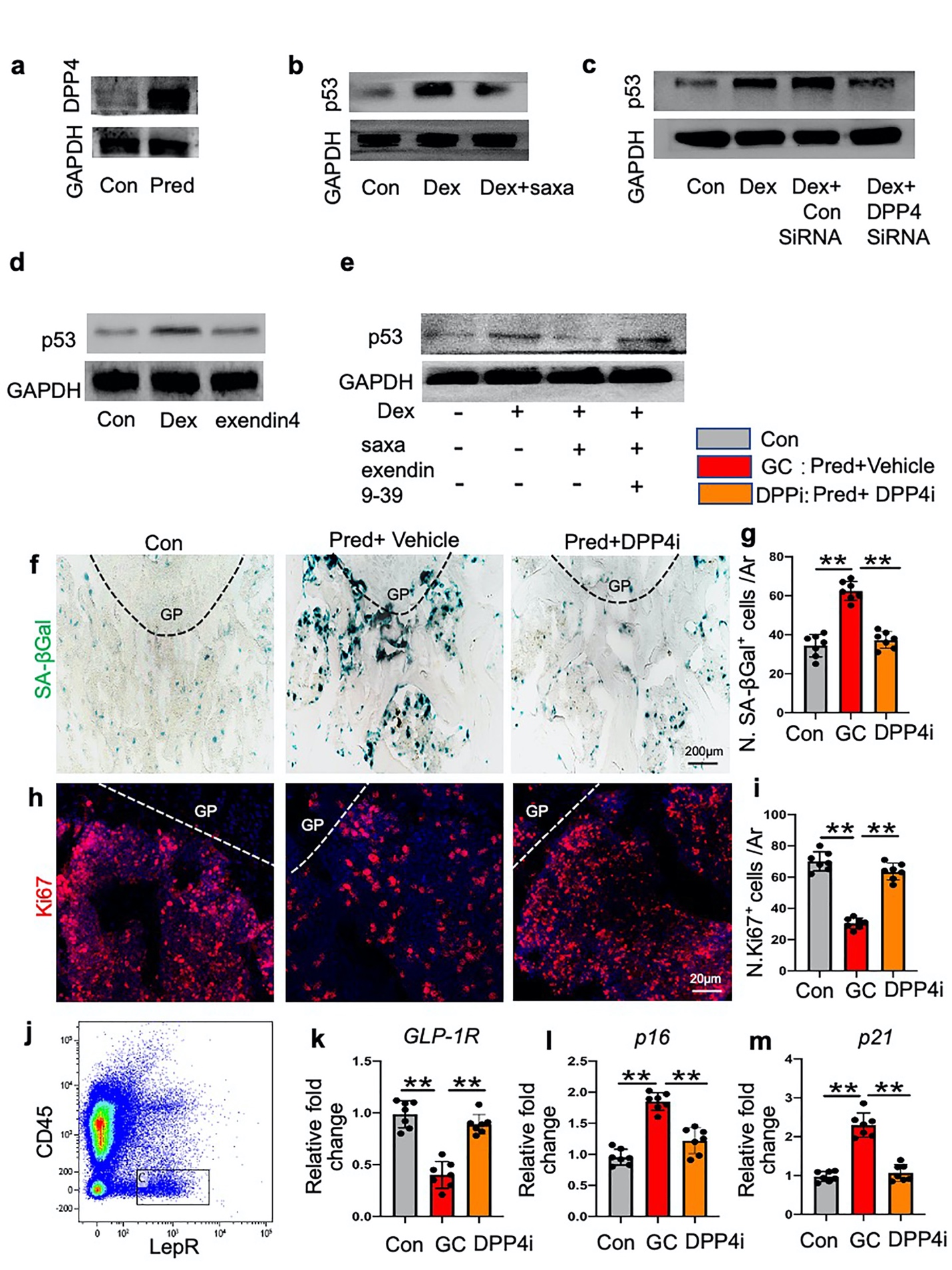
**

**Supplementary Fig. S6 The senescence effect of GCs on LepR^+^ BMSCs is controlled by DPP4-GLP1**

The protein level of DPP4 was increased after GC treatment (a). Saxagliptin was used as a pre-treatment 4 h before Dex (1 μM) treatment for 48 h. Western blot analysis of p53 in LepR^+^ cells (b). After pretreatment with DPP4 SiRNA for 72 h, the LepR^+^ cells were incubated with Dex for 48 h. The protein expression of p53 was analysed by western blot (c). The protein levels of p53 was decreased when using the GLP-1R agonist (exendin4) on LepR^+^ cells (d). The protein levels of p53 was increased in LepR^+^ cells when using the GLP-1R inhibitor (exendin 9-39) (e). Sitagliptin at a concentration of 4 g/kg mixed in chow was used in eight-week-old C57BL/6 male mice. Representative images of SA-βGal^+^ cells (blue) in metaphysis are shown in (f). Numbers of SA-βGal^+^ cells per trabecular bone surface (N. SA-βGal^+^ cells/ Ar) are shown in (g). Immunofluorescence staining. Immunostaining of Ki67 (red) using femural sections (h). Number of Ki67^+^ cells per trabecular bone surface (N. Ki67^+^ cells/ Ar) (i). Representative flow cytometry plots showing targeted cells sorted from bone marrow cells based on a CD45 negative and LepR positive sorting strategy (j). Quantitative real-time PCR analysis mRNA of GLP-1R (k), p16INK4a (l), and p21 (m). Seven mice per group were used and data are represented as mean ± s.e.m. GP, growth plate; Ar, tissue area. DAPI was used to stain nuclei blue. *p< 0.05, **p< 0.01; (One-way ANOVA).

**
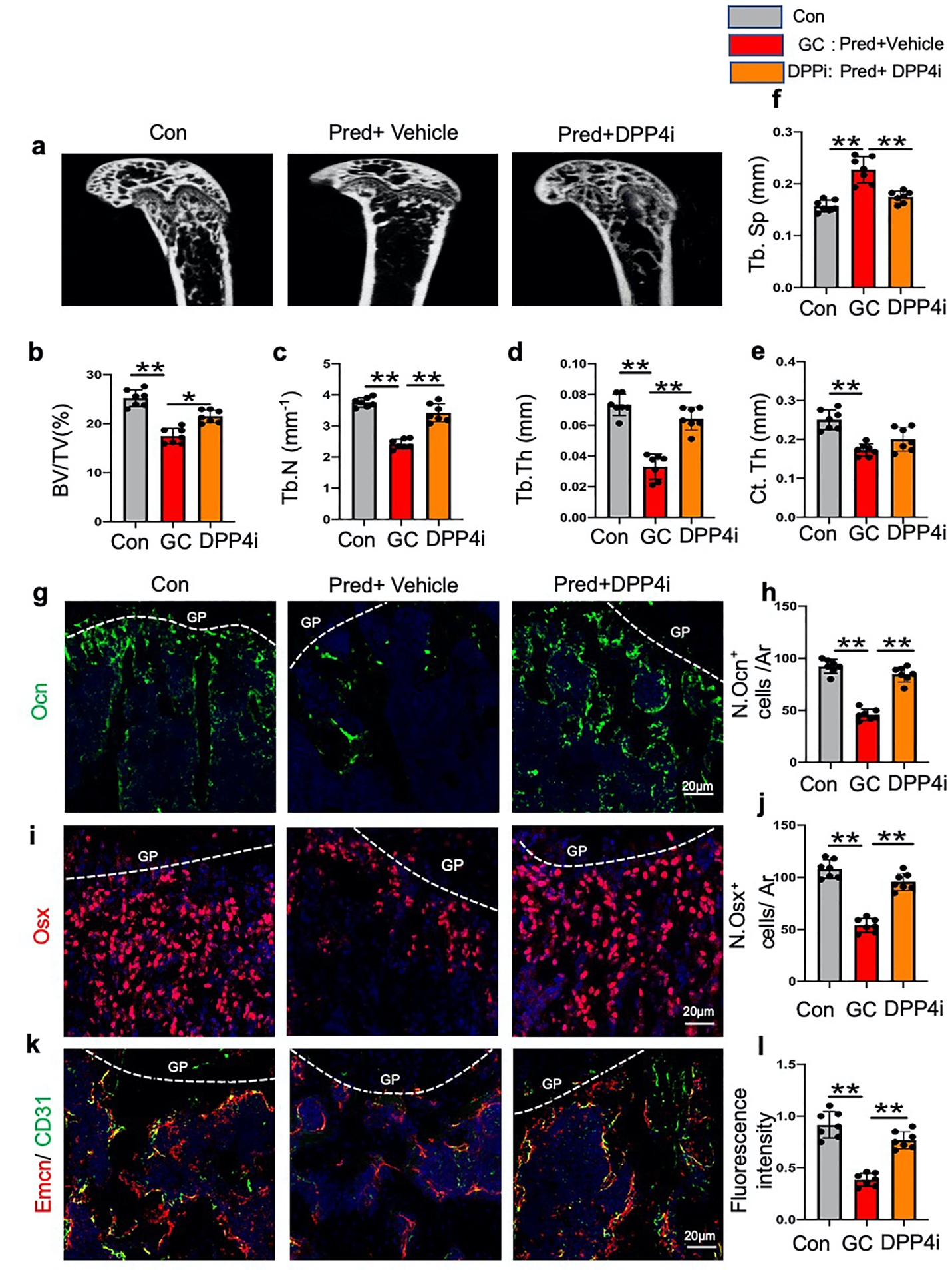
**

**Supplementary Fig. S7 Impact of DPP4 inhibition on bone metabolism**

The DPP4 inhibitor, sitagliptin, at a concentration of 4 g/kg mixed in chow was used (a-m). Representative micro-CT images of distal femurs (a, longitudinal sections). Quantitative analyses of bone volume/tissue volume (BV/TV) (b), trabecular number (Tb. N) (c), trabecular thickness (Tb. Th) (d), trabecular separation (Tb. Sp) (f) and cortical thickness (Ct. Th) (e).

Representative confocal images are shown in (g) and (i). Green: Ocn^+^cells; Red: Osx^+^cells; Blue: nuclear staining by DAPI. Quantification of Ocn^+^ (N. Ocn^+^ cells/ Ar) and Osx ^+^ cells (N. Osx ^+^ cells/ Ar) per trabecular bone surface are shown in (h) and (j), respectively. Representative images of H type vessels (double-immunofluorescence staining of endomucin [Emcn] [red] and CD31 [green]) are shown in (k). Quantification of relative fluorescence intensities in the metaphysis of long bones (l). GP, growth plate; Ar, tissue area. DAPI was used to stain nuclei blue. Seven mice per group. Data are represented as mean ± SD. *p< 0.05, **p< 0.01; (One-way ANOVA).

**
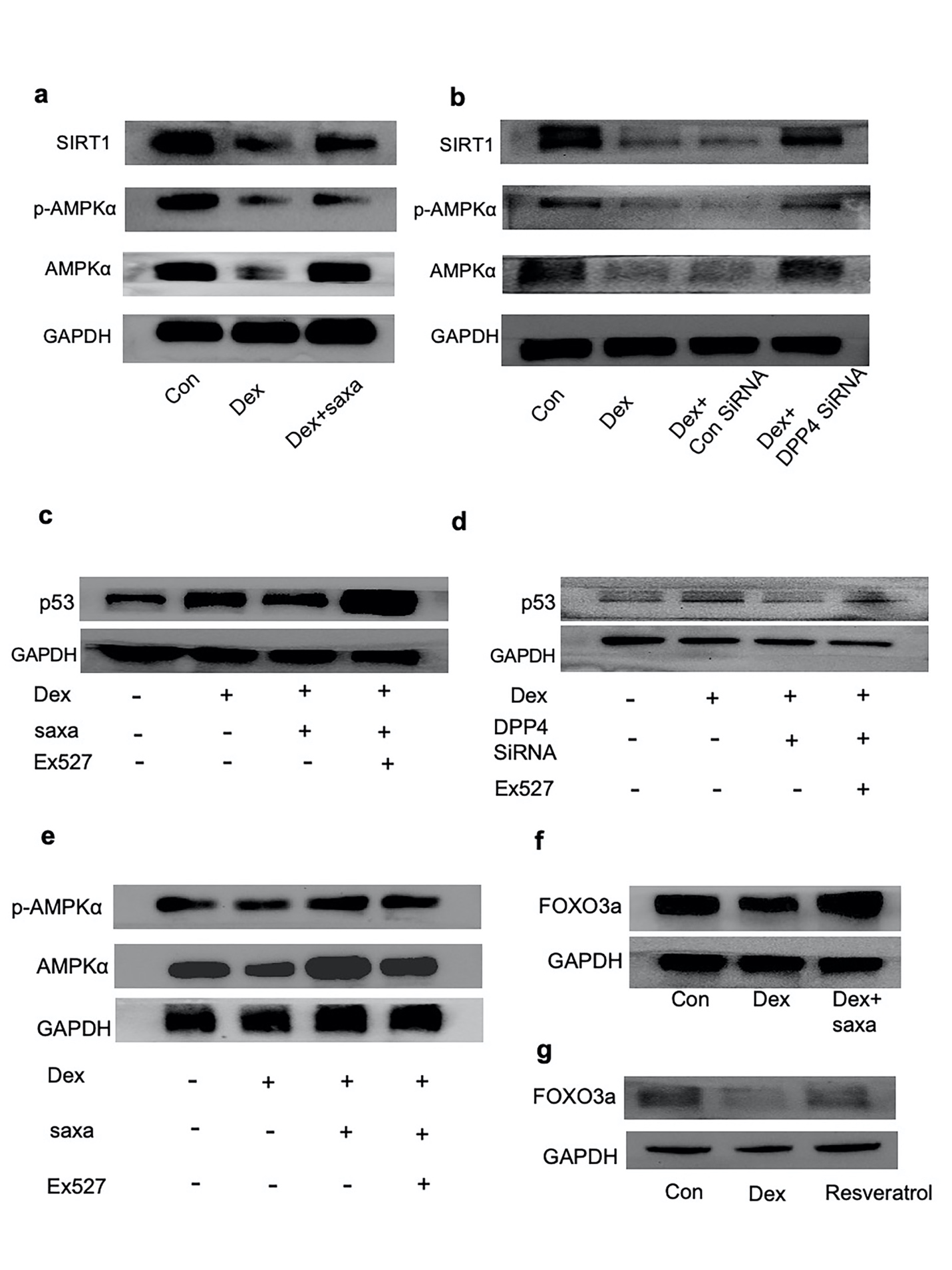
**

**Suplementary Fig. S8 The AMPKα/SIRT1/FOXO3a pathway is involved in GC-induced LepR^+^ MSC senescence**

Saxagliptin (saxa) was used as a pre-treatment 4 h before Dex (1 μM) treatment for 48 h. Western blot was performed to determine the levels of SIRT1, AMPKα, and p-AMPKα in LepR^+^ MSCs (a). After pre-treatment with DPP4 SiRNA for 72 h, the LepR^+^ MSCs were stimulated with Dex for 48 h. Western blot was performed to detect SIRT1, AMPKα, and p-AMPKα in LepR^+^ cells (b). LepR^+^ MSCs were treated with saxa for 4 h, with or without Ex527, before incubation with Dex for 48 h. The protein expression of p53 in LepR^+^ cells was mesuared by western blot (c). After pre-treatment with DPP4 SiRNA for 72 h, with or without Ex527, the LepR^+^ MSCs were incubated with Dex for 48 h. Immunoblot of p53 in LepR^+^ MSCs (d). LepR^+^ MSCs were treated with saxa for 4 h, with or without Ex527, before stimulation with Dex for 48 h. The protein expression and phosphorylation levels of AMPKα in LepR^+^ cells were mesuared by western blot (e). Immunoblot of FOXO3a in LepR^+^ cells treated with saxagliptin or treated with resveratrol (f, g).

**
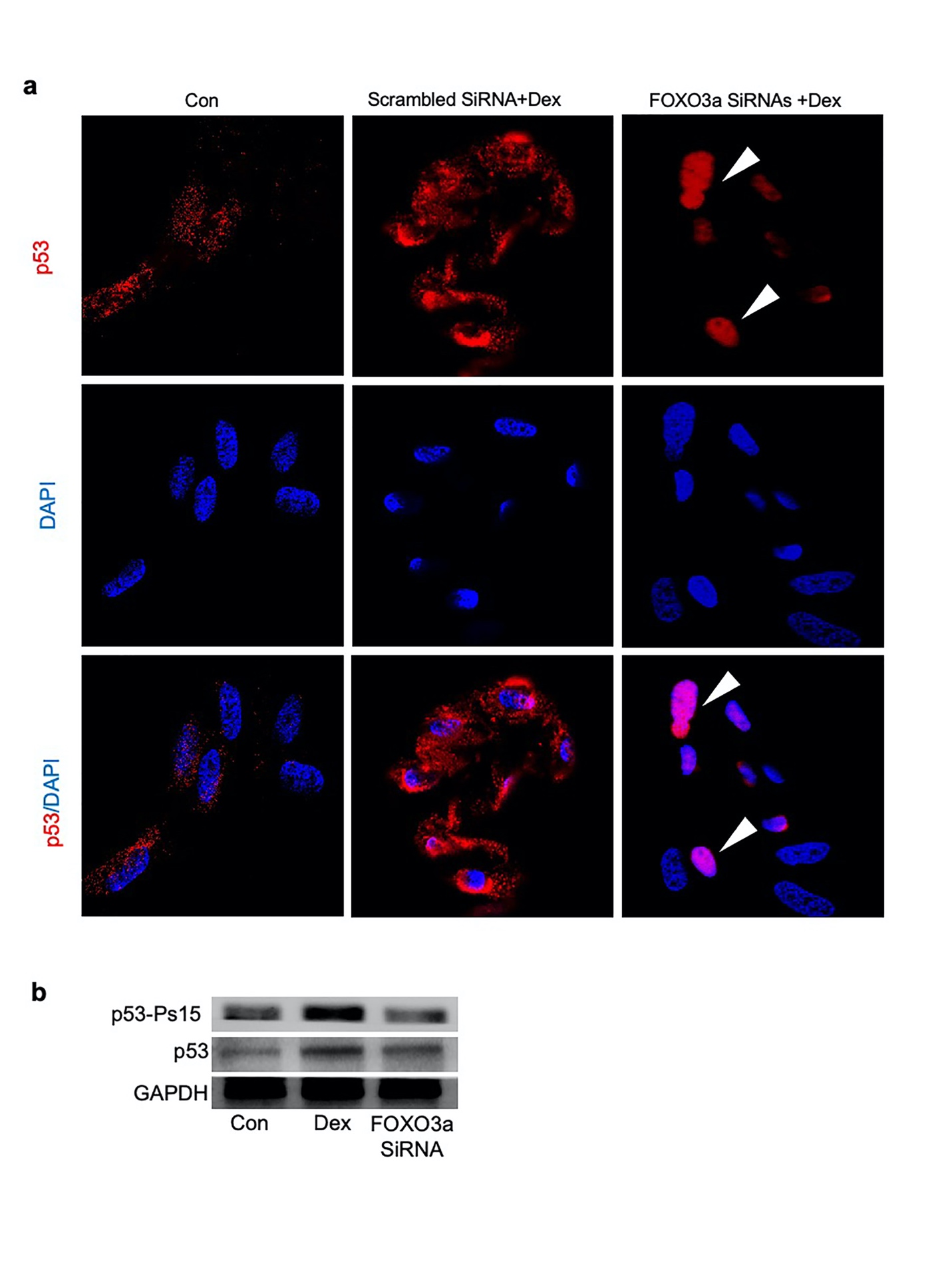
**

**Suplementary Fig. S9 FOXO3a regulates the subcellular translocation of p53**

Transfection of LepR^+^ MSCs with FOXO3a SiRNA or scrambled control siRNA (Scrambled SiRNA), then stimulated with Dex (1 μM). Cells were fixed and immunofluorescence staining was performed using an antibody against p53 (red) in (a). Arrow heads indicate nuclear translocation of p53. DAPI was used to stain nuclei blue. The protein expression of p53 and p53-pS15 in LepR^+^ cells with FOXO3a SiRNA (b).

**
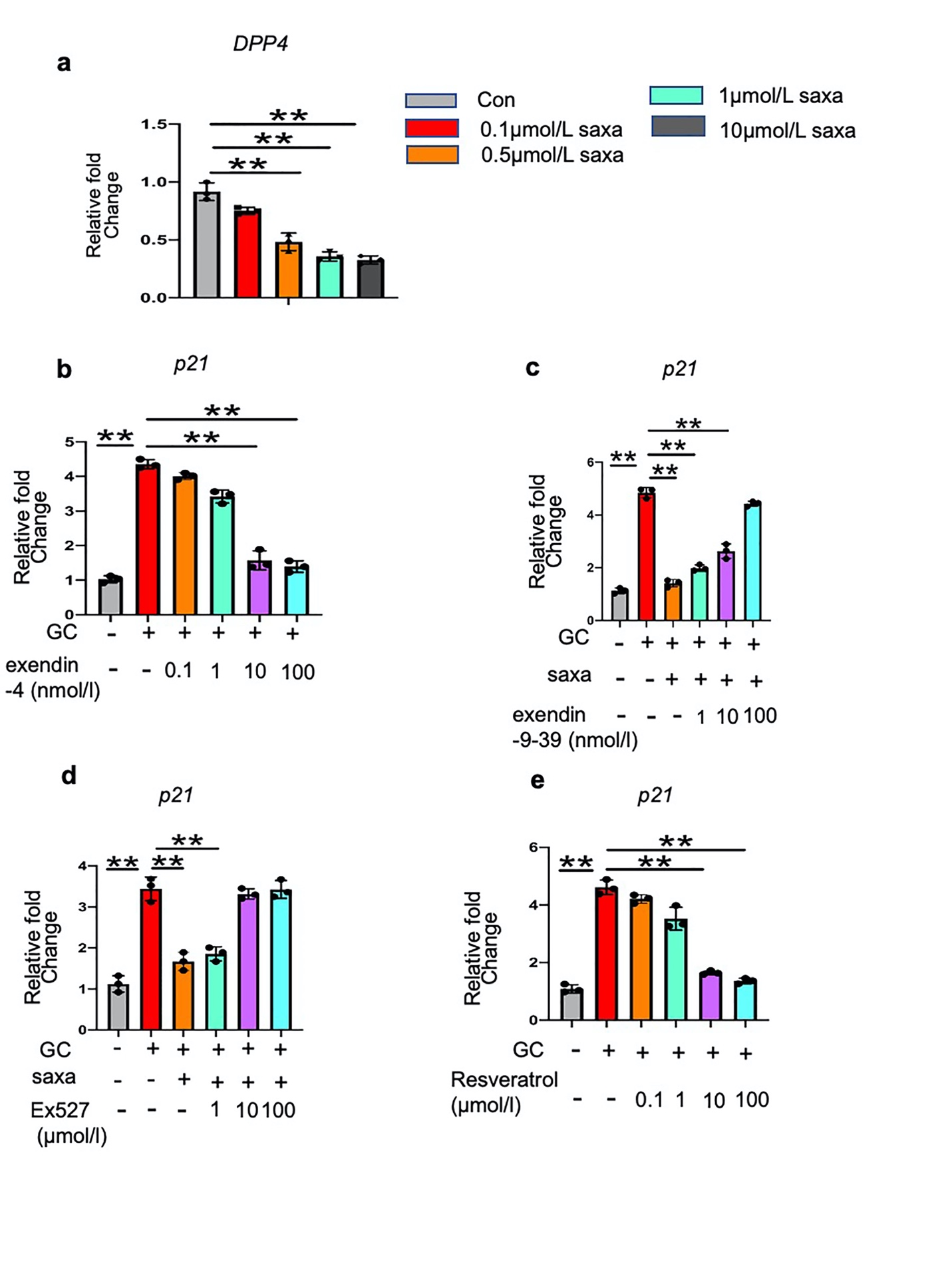
**

**Suplementary Fig. S10 Dose response analysis for the pharmacological agents**

Saxagliptin treatment (0.1–100 μM). Quantitative analysis of DPP4 activity (a). Quantitative RT-PCR analysis of the mRNA of p21 in LepR^+^ cells treated with exendin-4 and exendin 9–39 (b, c). Quantitative RT-PCR was performed to assess the mRNA levels of p21 in LepR^+^ MSCs treated with Ex527 or resveratrol (d, e). Data are expressed as mean ± SD. *p< 0.05, **p< 0.01; (One-way ANOVA).
